# Ataxia-Telangiectasia: A Case Report and a Brief Review

**DOI:** 10.7759/cureus.39346

**Published:** 2023-05-22

**Authors:** Abulkalam A Sirajwala, Shahin Khan, Vaishnavi M Rathod, Vishwa C Gevariya, Jay R Jansari, Yash M Patel

**Affiliations:** 1 Department of General Medicine, Sir Sayajirao General Hospital and Medical College Baroda, Vadodara, IND

**Keywords:** alpha-fetoprotein, a.t.m gene, cerebellum degeneration, dystonia, telangiectasia, ataxia

## Abstract

Ataxia-telangiectasia (A-T) is a rare inherited syndrome that primarily presents as ataxia due to cerebellar involvement and dilated capillaries in the oculocutaneous region. But many more serious complications of the condition exist, due to which both the quality and length of life are severely affected. Some of these include opportunistic infections due to an abnormal immune system, various malignancies, and an increased sensitivity to ionizing radiation. Due to the involvement of multiple systems in the body, diagnosis of this condition could be tricky as it may manifest with uncommon signs like dystonic head movements seen in our case. We have presented the case of a 16-year-old male born out of a consanguineous marriage, with the major symptoms of walking difficulties, frequent falls, and jerky movements of the head. Similar or related complaints had been noted in the past in his siblings. Laboratory investigations revealed elevated levels of serum alpha-fetoprotein, while the confirmatory diagnosis was made by genetic testing of the ataxia-telangiectasia mutated (ATM) gene. The patient was treated with amantadine and clonazepam, along with speech therapy, but the prognosis remained poor due to the lack of curative treatment for A-T.

## Introduction

Ataxia-telangiectasia (A-T) is a rare, progressive neurodegenerative inherited condition that involves multiple systems and is usually diagnosed in early childhood. The presenting complaints could be ataxia, oculocutaneous telangiectasia, immunodeficiency, frequent pulmonary infections, and certain cancers [1]. The ataxia-telangiectasia mutated (ATM) gene, located on chromosome 11q22-23, is affected in this condition which is inherited in an autosomal recessive manner and results in diffuse atrophy of the cerebellum due to the loss of Purkinje fibers. Children with A-T can develop various complications due to an abnormal immune system and increased sensitivity to ionizing radiation. Symptomatic and supportive management of the symptoms and complications, early screening for cancers, and limiting exposure to radiation can be done, but there is no cure for this condition [2]. Genetic counseling should be done to educate the parents of affected children and to identify A-T in the antenatal period. We have presented a rare case of a 16-year-old male who came to the hospital with complaints of ataxia and ocular telangiectasia with a positive family history of A-T. The patient also had complaints of dystonic neck movements, which is an uncommon finding in such a younger age group.

## Case presentation

**Figure 1:**
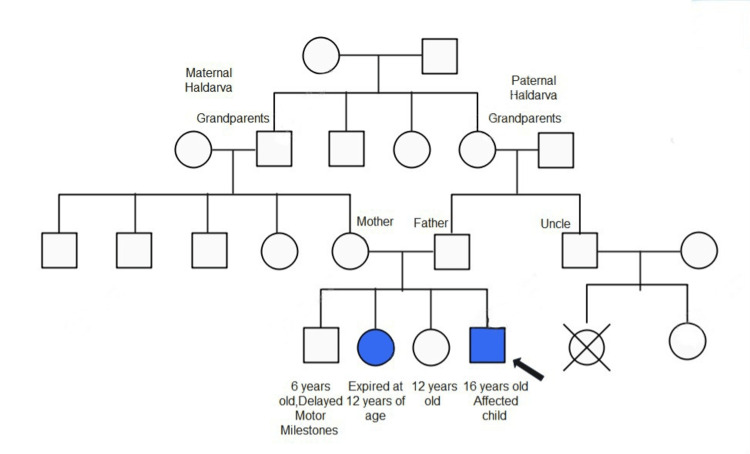
Pedigree chart of the family representing consanguineous marriage and the affected children

A 16-year-old male presented with complaints of difficulty in walking, frequent falls, and jerky movements of the head on the left side at rest. Difficulty in movements started at the age of two-three years and gradually progressed to the current state, where he requires support to walk. The patient had no complaints of fever, cough, difficulty in breathing, neck rigidity, convulsions, blurring of vision, or sensory deficit in the past. Difficulty in crawling, creeping, and sitting had been noted during his childhood. There was a history of consanguineous marriage between his parents (Figure 1).

Similar complaints were present in the patient's sister, who was diagnosed with A-T and died at the age of 12 due to bronchiectasis. A brother of the patient, who had a history of delayed motor milestones, died at the age of six years.

**Figure 2:**
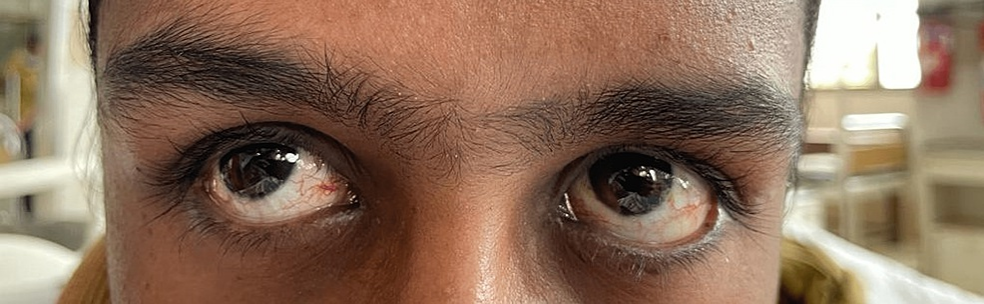
Ocular telangiectasia in both the eyes

On examination, cardiovascular and respiratory findings were normal. Some notable findings included ocular telangiectasia, oculomotor apraxia, and ataxic speech (Figure 2). Dystonic neck movements, which are difficult to find at such a young age, were also present. Hypotonia in all four limbs was seen, along with action tremors in both hands. There was an absence of deep tendon reflexes with preserved superficial reflexes. The involvement of the cerebellum was determined by a wide-based gait, dysdiadochokinesia, nystagmus, positive knee-heel test, positive finger-nose coordination, and positive finger-to-finger test, and it was also supported by a positive Romberg's sign. The differential diagnoses following the examination included ataxia with spastic paraplegia and ataxia with dystonia, with a localized lesion in the cerebellum.

**Figure 3:**
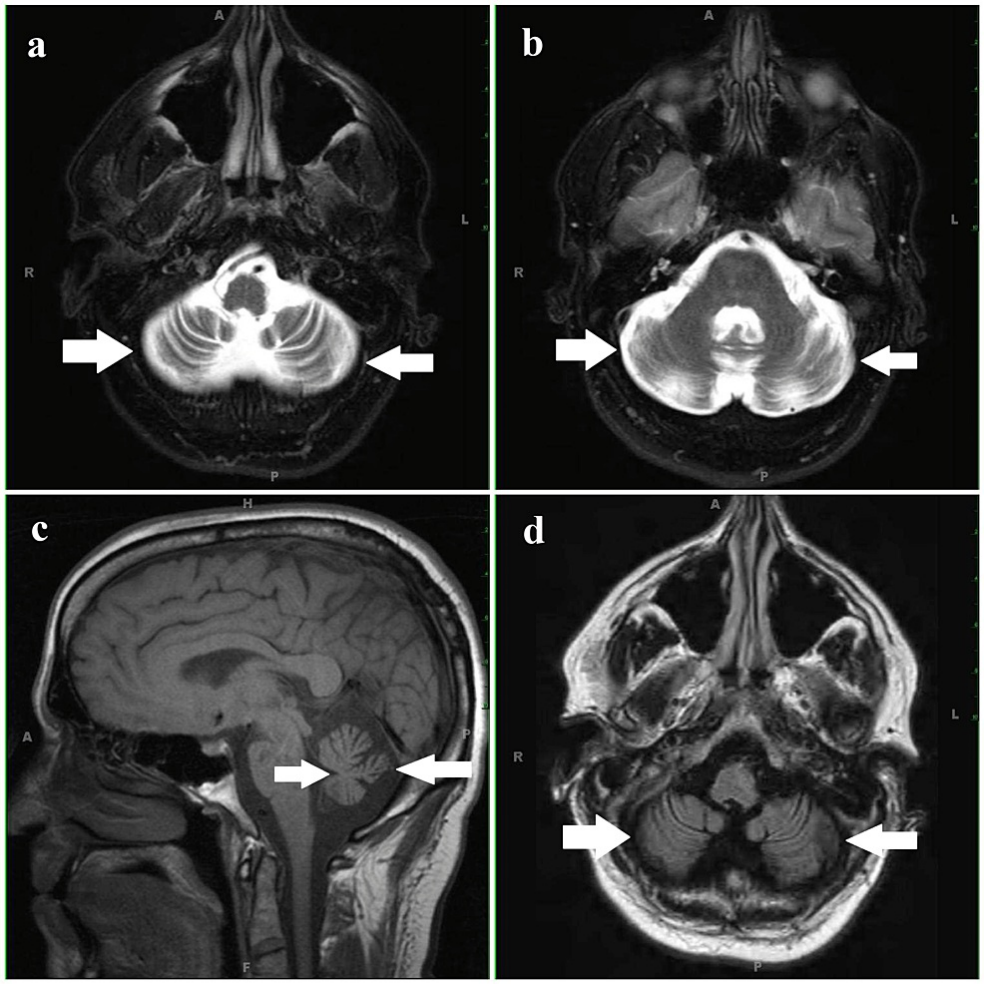
MRI of the brain a, b: MRI of the brain (T1) axial section with contrast showing cerebellar degeneration with the prominence of adjacent subarachnoid space. c: MRI of the brain (T1) sagittal section showing cerebellar degeneration. d: MRI of the rain (T1) axial section without contrast showing cerebellar degeneration with the prominence of adjacent subarachnoid space.

During routine laboratory investigations, complete blood count (CBC), serum B12 level, vitamin E level, and electrolytes were normal, while serum alpha-fetoprotein (AFP) was elevated to 460.7 ng/ml. The chest X-ray was not suggestive of any infection. The lymphocyte count and serum immunoglobulin were towards the lower margin of the normal range. An MRI of the rain (plain and with contrast) was done, which revealed the dilatation of bilateral cerebellar sulci with dilatation of subarachnoid spaces, inferring the patient had bilateral cerebellar degeneration (Figure 3). A diagnosis of A-T was confirmed by the detection of a frameshift mutation after performing whole genome sequencing. The patient was managed symptomatically by providing support for walking. Amantadine (100 mg) and clonazepam (10.5 mg) were prescribed along with other supportive management, and the patient was sent for speech therapy. The parents of the patient were counseled about the risk of recurrent lower respiratory tract infections (LRTI).

## Discussion

A-T, also known as Louis-Bar syndrome, belongs to the group of hereditary ataxias that is inherited in an autosomal recessive pattern. In the present case, two children were diagnosed, and one was a suspected case of A-T out of a total of four siblings. This is unusual for a disease that is inherited in an autosomal recessive pattern. It involves multiple systems in the body and results in progressive cerebellar ataxia, oculocutaneous telangiectasias, frequent pulmonary infections, immunodeficiency, increased risk of certain malignancies, and radiation sensitivity. The prevalence of the disease is between one in 40,000 to one in 100,00 [1]. A mutation in the ATM** **gene located on chromosome 11q22-23 is known to be the cause [2]. On neuroimaging, diffuse progressive degeneration of different parts of the cerebellum has been seen. The loss of Purkinje fibers in the cerebellum and malfunction of the other neuronal cells are attributed to the degeneration [1]. 

The clinical features differ notably in the time of manifestation, incidence, and progression among children, which makes the diagnosis difficult [3]. The different presentations in children are categorized as classic/typical/early-onset in case of the presence of all characteristic features and as variant/atypical/adult-onset in case of the presence of only some characteristic features, or less severe or late-onset of features [1]. In typical presentations, ataxia is recognized in early childhood, during the time when a child learns to sit and stand. The child may start walking at a normal age, but their balance worsens as the child grows. It gets tough for the child to stand or sit still, and often starts swinging from side to side. In the school years, ataxia progresses to such a state that the child walks by holding walls and doors and eventually requires a wheelchair, as seen in our case [1,4]. Telangiectasia usually occurs at the age of 5-8 years, but in some cases, it can be present at birth or develop during the teenage years. The bulbar conjunctiva is a common site for telangiectasias, but it can later develop on other body parts such as the nares or pinna [4].

Visual acuity is not affected, but there are abnormal eye movements and impaired fixation of images, which cause difficulty in reading. Abnormal eye movements such as oculomotor apraxia, nystagmus, convergence, saccades, and saccadic intrusions can be observed [5]. The children may develop dysarthria, myoclonic jerks, difficulty with fine motor movements, tremors, chorea, and athetosis, which hampers daily activities [1,6]. Loss of tendon reflexes from distal to proximal is also common in A-T, which is explained by progressive peripheral sensory and motor neuropathy [1,3]. Any abnormalities in the immune system, especially reduced immunoglobulins, result in frequent pneumonia, bronchiectasis, and interstitial lung disease (ILD) [1]. There is an increase in the risk of cancer among patients with A-T. In classical patients, lymphomas and leukemias are the most common, while solid tumors of the breast and liver are also seen in adults [1,7]. There is an increased sensitivity to ionizing radiation (X-rays and gamma rays) among patients with A-T. Even diagnostic doses of X-ray and radiation therapy are harmful to patients and hence, should be limited to use only when necessary and in balanced doses [1,8]. 

The absence of awareness of A-T and the late onset of telangiectasia hampers the early diagnosis of the condition. The presence of multiple complaints and abnormal laboratory findings is used to make the diagnosis. Abnormal laboratory findings include increased AFP, as seen in our case, reduced immunoglobulin, lymphopenia, X-ray-induced chromosomal breaks, and reduced survival after exposure to ionizing radiation of cultured lymphocytes and fibroblasts [1]. The absence or deficiency of ATM protein in cultured cell lines established from lymphocytes or skin biopsies confirms the diagnosis of A-T, but it is not always required [9]. Certain disorders such as ataxia-telangiectasia-like disorder (ATLD), ataxia oculomotor apraxia type 1 (AOA1), type 2 (AOA2), and cerebellar ataxia with retained reflexes because of similar clinical presentations, and the presence of a positive family history can be confused with A-T [10]. A combination of clinical symptoms and specific laboratory findings is the key to differentiating A-T from other conditions [1].

A-T has no definitive cure available [11]. The management of A-T involves symptomatic and supportive treatment. Multidisciplinary treatment and potential therapy by using anti-oxidants and mutation-targeted approaches have been recommended in some literature [11,12]. For parents with an affected child, genetic counseling is of utmost importance to educate them about the condition, its genetics, progression, consequences, and how antenatal testing can make a difference by identifying the condition before birth.

## Conclusions

Ataxia-telangiectasia is a rare autosomal recessive disorder that is often tricky to diagnose owing to an unpredictable presentation due to the involvement of multiple systems. This could lead to uncommon signs and symptoms, such as the dystonic head movements seen in our case. Genetic counseling of the affected child's parents is of utmost importance as there is a probability of having other affected children in the future too. The poor prognosis is compounded by an increased susceptibility to some infections and cancers. Active measures should be taken for early diagnosis, especially in cases with a family history of A-T. The treatment is symptomatic and supportive, but the outcomes are generally poor. To summarize, a young patient presenting with ataxia should be enquired for a family history of similar complaints and thoroughly examined for the presence of telangiectasias, especially in the ocular region. Ataxia associated with immunodeficiency leading to recurrent respiratory infections or associated with malignancies should also raise suspicion about the possibility of A-T.
